# *Cuscuta* species: Model organisms for haustorium development in stem holoparasitic plants

**DOI:** 10.3389/fpls.2022.1086384

**Published:** 2022-12-12

**Authors:** Min-Yao Jhu, Neelima R. Sinha

**Affiliations:** ^1^ Crop Science Centre, Department of Plant Sciences, University of Cambridge, Cambridge, United Kingdom; ^2^ Department of Plant Biology, University of California, Davis, CA, United States

**Keywords:** *Cuscuta*, parasitic plants, haustorium, model organisms, parasitism, holoparasite, organ development, organogenesis

## Abstract

Parasitic plants are notorious for causing serious agricultural losses in many countries. Specialized intrusive organs, haustoria, confer on parasitic plants the ability to acquire water and nutrients from their host plants. Investigating the mechanism involved in haustorium development not only reveals the fascinating mystery of how autotrophic plants evolved parasitism but also provides the foundation for developing more effective methods to control the agricultural damage caused by parasitic plants. *Cuscuta* species, also known as dodders, are one of the most well-known and widely spread stem holoparasitic plants. Although progress has been made recently in understanding the evolution and development of haustoria in root parasitic plants, more and more studies indicate that the behaviors between root and stem haustorium formation are distinct, and the mechanisms involved in the formation of these organs remain largely unknown. Unlike most endoparasites and root holoparasitic plants, which have high host-specificity and self- or kin-recognition to avoid forming haustoria on themselves or closely related species, auto-parasitism and hyper-parasitism are commonly observed among *Cuscuta* species. In this review, we summarize the current understanding of haustorium development in dodders and the unique characteristics of their parasitizing behaviors. We also outline the advantages of using *Cuscuta* species as model organisms for haustorium development in stem holoparasitic plants, the current unknown mysteries and limitations in the *Cuscuta* system, and potential future research directions to overcome these challenges.

## 1 Introduction

Plants have often been defined as autotrophic eukaryotic photosynthetic organisms. Plants have chlorophyll, which enables them to convert inorganic carbons into carbohydrates utilizing light energy. However, parasitic plants are exceptions to this definition. They evolved to form a specialized organ, the haustorium, which allows parasitic plants to obtain water and nutrients from their host plants (Yoshida et al., 2016; Kokla and Melnyk, 2018). Depending on the haustorium attachment position on their host, parasitic plants are generally categorized as stem or root parasites. Based on their host dependency, parasitic plants are classified as hemiparasites or holoparasites. Holoparasitic plants have mostly lost their photosynthetic ability and need to rely entirely on their hosts to provide water and nutrients. Therefore, haustorium formation has been considered an essential element for plant parasitism (Yoshida et al., 2016; Kokla and Melnyk, 2018). Investigating the mechanism involved in haustorium development reveals how autotrophic plants evolved to acquire heterotrophic lifestyles. Our evolutionary developmental knowledge of haustorium formation also provides the foundation for developing more effective methods to control the agricultural damage caused by parasitic plants.

*Cuscuta* species (dodders) are the most well-known and widely spread stem holoparasitic plants. The *Cuscuta* genus comprises about 200 species and is the only genus that evolved parasitism in the Convolvulaceae (Yuncker, 1932). *Cuscuta* plants have degenerated roots and leaves and have stems that can coil around their host in a counterclockwise manner. Depending on the species and the growth conditions, *Cuscuta* stems are orange-yellow, yellow, or greenish-yellow because they only have a meagre amount of chlorophyll. Several previous studies on genomes of *Cuscuta* species indicate that *Cuscuta* species have lost some genes needed for efficient photosynthetic activity (McNeal et al., 2007; Braukmann et al., 2013; Sun et al., 2018; Vogel et al., 2018), which can be considered evidence for transitioning from hemiparasites to holoparasites. Therefore, obtaining water and nutrients from their host is the top priority for their survival. Unlike root parasitic plants that mostly depend on haustorium-inducing factors (HIFs) (Yoshida et al., 2016), the *Cuscuta* haustorium induction is also regulated by environmental signals such as light signals (Furuhashi et al., 2011). These unique lifestyles and morphological characteristics make *Cuscuta* species a good system to study how autotrophic plants evolved parasitism by attaching onto above-ground organs of their host plants.

Aside from the opportunities they present for investigating evo-devo mysteries, *Cuscuta* plants are the focus of intensive research since they cause massive agricultural losses. Several *Cuscuta *species are listed in multiple countries’ noxious weed lists (Holm et al., 1997) because they parasitize a wide range of important vegetable and fruit crops, and ornamental plants (Lanini and Kogan, 2005). Comparing with *Striga* species that mostly target monocot hosts, the host plants for *Cuscuta* species are primarily eudicots, but a few monocot crops can also reportedly be attacked by *Cuscuta *species (Lanini and Kogan, 2005). *Cuscuta* species are parasites on 25 crops in at least 55 countries (Holm et al., 1997; Lanini and Kogan, 2005). If left uncontrolled, *Cuscuta* growth will decrease host nutrient status and lead to reduced stand, canopy, biomass and fruit weight (Musselman, 1987; Lanini and Kogan, 2005). Yield reductions of 50–72% in tomatoes and 70–90% in carrots have been reported (Lanini and Kogan, 2005; Mishra et al., 2006). In temperate climates, yield losses up to 80–100% have been reported in cranberry in the US due to *Cuscuta*  (Devlin and Deubert, 1980). In California alone, over 12,000 ha. are affected by *Cuscuta*  (Lanini and Kogan, 2005). Even when crop rotation is practised, *Cuscuta* is hard to eradicate due to long-term seed viability. Therefore, a better understanding of the mechanisms involved in *Cuscuta* haustorium development is required to build more effective control strategies to stop the agricultural damages brought on by *Cuscuta*. During the past decade, progress has been made in understanding the interaction between *Cuscuta* species and their hosts, especially on the mechanism involved in resistance responses against *Cuscuta* species (Kaiser et al., 2015; Jhu and Sinha, 2022), and the signal exchange and horizontal gene transfer between *Cuscuta* plants and their hosts (Kim and Westwood, 2015; Wu, 2018; Yang et al., 2019; Lin et al., 2022). These aspects have been reviewed recently (Kaiser et al., 2015; Kim and Westwood, 2015; Wu, 2018; Jhu and Sinha, 2022). On the other hand, although review articles cover some specific aspects of *Cuscuta* haustorium development and parasitism behaviours (Yoshida et al., 2016; Shimizu and Aoki, 2019), several recent discoveries in the field have not been systematically reviewed yet.

This review summarises our current knowledge of the mechanism involved in the four stages of *Cuscuta* haustorium development, including the initiation, adhesion, penetration, and vascular connection. We discuss the unique characteristics of the parasitizing behaviours in *Cuscuta* species compared with other well-studied root parasitic plants. These unique features are compelling reasons to use *Cuscuta* species as model organisms for studying haustorium development in stem holoparasitic plants. We outline the advantages, unknown mysteries, and current limitations of the *Cuscuta* system. We also propose potential models and directions that might overcome these challenges.

## 2 Before haustorium formation: Finding their hosts

**Figure 1:**
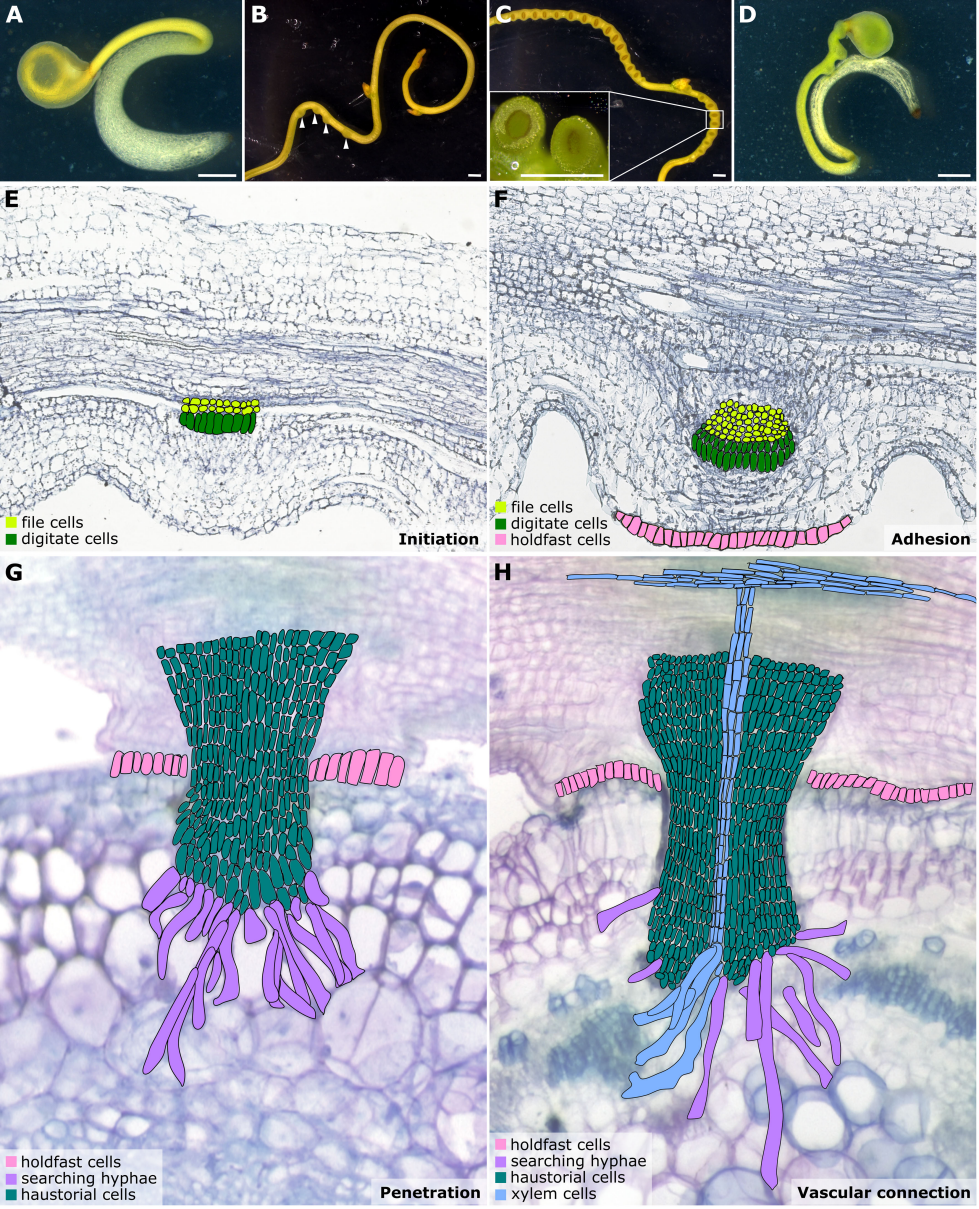
Illustrations of *Cuscuta* developmental stages and haustorium organogenesis. **(A–D)** *Cuscuta campestris* seedlings and strands with prehaustoria and haustoria at different developmental stages. **(A)** A *Cuscuta campestris* seedling. **(B)** A *Cuscuta campestris* strand with prehaustoria. White arrowheads indicate prehaustoria. **(C)** Haustoria with adhesive disks on a *Cuscuta campestris* strand. Adhesive disks are enlarged in the subfigure. **(D)** A *Cuscuta campestris* seedling autoparasitizes itself. **(A–D)** Scale bar = 1 mm. **(E–H)** Four major haustorium developmental phases in *Cuscuta* species. **(E)** Initiation phase: The prehaustorium structure appears because the disc-like meristem emerges in the inner cortex region of the *Cuscuta* stem. The prehaustorium primordia consist of file cells and digitate cells. **(F)** Adhesion phase: The epidermal cells of *Cuscuta* stems divide and differentiate into holdfast cells, which comprise the adhesive disk to secure the adhesion of haustorial attachments. **(G)** Penetration phase: The cortex-originated inner haustorial cells penetrate through the epidermis and cortex of their host tissues. At the tip of the haustorium, the inner haustorial cells elongate and differentiate into searching hyphae, which start invading through the apoplastic region of host cortex tissues and searching for host vasculature. **(H)** Vascular connection phase: Establishing a successful vascular connection is the final stage of haustorium organogenesis. If the searching hyphae reach host xylem cells or phloem cells, they obtain xylem or phloem identity and differentiate into xylem- or phloem-conductive elements, respectively. Part of this figure **(B, C)** is modified from images in a previously published paper Jhu et al., 2021 with new information added.

*Cuscuta* seeds are about 1 mm in size and cannot store sufficient nutrients to support seedling growth for longer than one week (**Figures 1A–D**). Therefore, finding a suitable host and forming successful haustorial attachments within a few days post-germination are important for seedling survival. Previous research indicates that *Cuscuta* seedlings can detect the volatiles released by their hosts and use these chemical cues to locate their preferred hosts (Runyon et al., 2006). In addition to airborne chemical signals, previous studies also indicate that the high far-red light relative to red light plays a vital role in controlling *Cuscuta* parasitism and growth directions (Furuhashi et al., 1995; Orr et al., 1996; Tada et al., 1996). This signal might help *Cuscuta* find green and healthy plants as their hosts (Orr et al., 1996). Blue light has been reported to be essential for the twining behavior of *Cuscuta* stems (Furuhashi et al., 1995; Furuhashi et al., 2021), which is tightly associated with the following haustorium induction phase.

## 3 Cuscuta haustorium organogenesis

Haustorium organogenesis in *Cuscuta* species has been well-reviewed (Yoshida et al., 2016; Kokla and Melnyk, 2018; Shimizu and Aoki, 2019). However, the developmental stages are reported differently among references and diverse names are used to refer to the same stage, leading to some confusion. Here, we aim to summarise the *Cuscuta* haustorium organogenesis process and standardize the vocabulary used. Based on cell types and morphological characteristics, we classified haustorium organogenesis in *Cuscuta* species into four development stages: the initiation phase, adhesion phase, penetration phase, and vascular connection phase (**Figures 1E–H**).

### 3.1 Initiation phase (prehaustorium)

Once *Cuscuta* plants find their hosts and successfully coil around them, the next step is the start of haustorium initiation and formation of the prehaustorium, which is the immature haustorial structure seen prior to penetration of the host tissues (Yoshida et al., 2016). A group of cortical cells begin to accumulate starch-containing amyloplasts and enlarged nuclei. These cells are identified as the initial cells, which will then dedifferentiate and develop into haustorial meristem cells. The disc-like meristem that emerges in the inner cortex of the *Cuscuta* stem is the prehaustorium primordia, consisting of two types of cells: file and digitate cells (**Figure 1E**) (Lee, 2007). This primordium is also known as the “endophyte primordium” because it develops into an “endophyte.” An endophyte refers to the inner haustorial structure that grows into the host tissues during the penetration phase (Kuijt, 1977; Lee, 2007).

**Figure 2:**
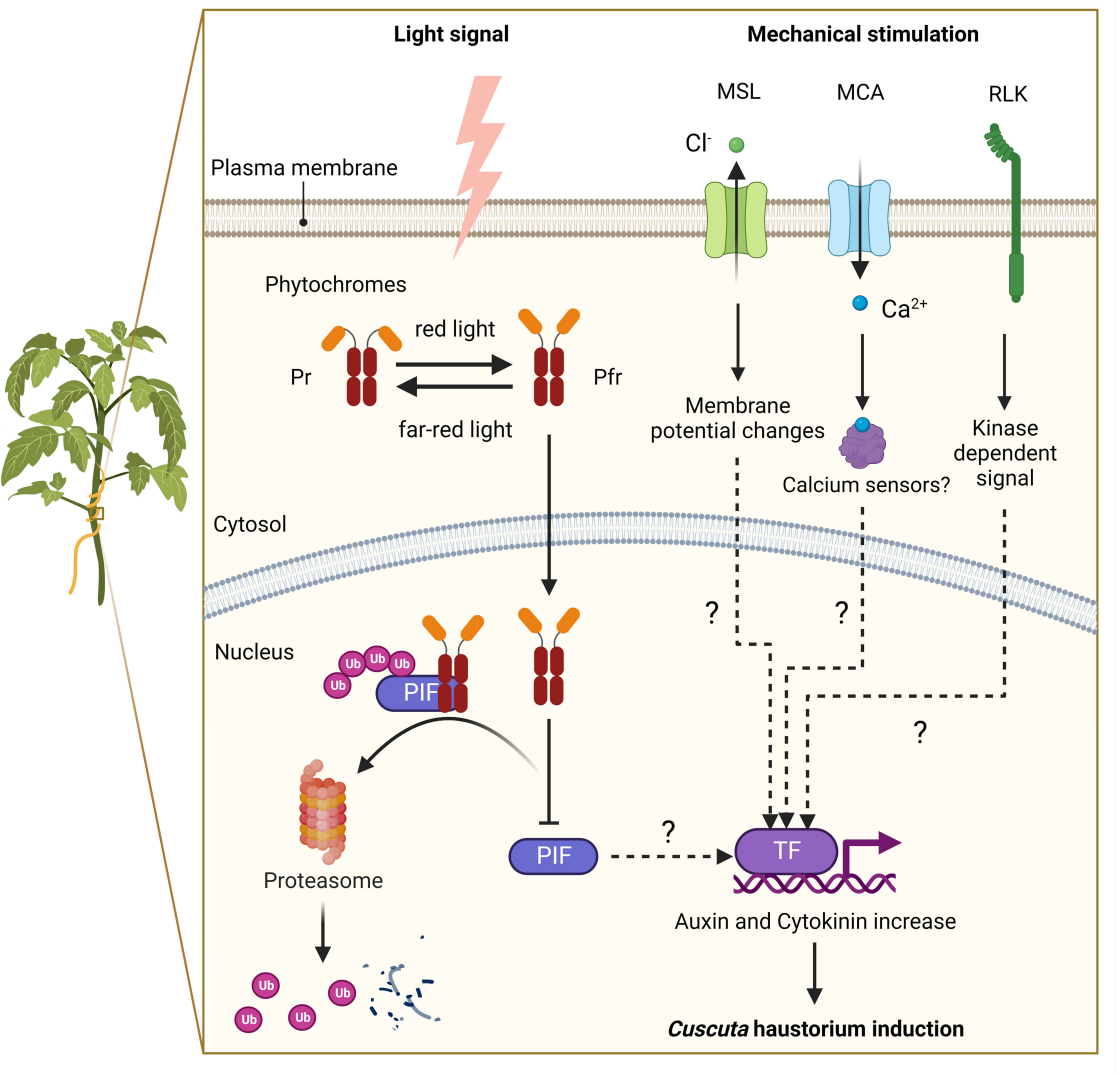
A putative model of signalling pathways for haustorium induction in *Cuscuta* species. Far-red light signal and mechanical stimulation are known to be the two major factors in inducing *Cuscuta* haustorium development. *Cuscuta* species likely have adopted far-red light signaling transduction and use phytochromes to regulate haustorium initiation. In high red-light conditions, phytochromes are converted from inactive form (Pr) to active form (Pfr), which will translocate from the cytosol into the nucleus and interact with phytochrome interacting factors (PIFs). PIFs are transcription factors, which are likely to regulate the downstream genes involved in hormone biosynthesis or transport. Interacting with Pfr phytochromes leads to PIFs phosphorylation and subsequent degradation. In high far-red light conditions, phytochromes are in the Pr inactive form and cannot enter the nucleus. Therefore, PIFs are released from repression and activate genes involved in haustorium initiation. Mechanosensing is another required element for *Cuscuta* haustorium development. A physical contact signal with hosts might activate mechanosensory proteins such as ion channels and receptor-like kinases. Mechanosensory ion channels elicit cytosolic Ca^2+^-dependent signaling and regulate downstream gene expression via unknown mechanisms. Receptor-like kinases trigger protein kinase cascades and then influence downstream gene transcription, which can lead to hormone status changes and haustorium induction.

Based on previous studies, the two primary triggers for *Cuscuta* haustorium initiation are the far-red light signal and mechanical stimulation (Tada et al., 1996; Furuhashi et al., 2011) (**Figure 2**). Upon receiving these light signals and physical contacts, the prehaustorium structure starts to develop. *Cuscuta* species probably employ phytochromes to sense the change in red light and far-red light ratio and adopt phototropism signaling transduction to control the initiation of haustoria. In Arabidopsis, phytochromes are transformed from their inactive form (Pr) to their active form (Pfr) in high red-light environments. Pfr then translocates from the cytoplasm into the nucleus, where Pfr interacts with phytochrome interaction factors (PIFs). As a result of their interaction, PIFs are phosphorylated and then degraded. PIFs are transcription factors that regulate the downstream gene expression involved in skotomorphogenesis and photomorphogenesis in Arabidopsis. *Cuscuta* species likely co-opt similar signaling pathways and use phytochromes to control the genes involved in hormone transport or biosynthesis (**Figure 2**) (Furuhashi et al., 1997; Furuhashi et al., 2011; Furuhashi et al., 2021; Pan et al., 2022). A previous study indicates that strong far-red light or low red to far-red ratios (low red light and high far-red light mixture condition) promoted *Cuscuta* stem coiling and haustorium formation (Furuhashi et al., 1997; Haidar and Orr, 1999). The results of *in vivo* quantification of the percentage of phytochrome status also showed that a maximum number of prehaustoria were produced when a low percentage of phytochrome is in Pfr form (Haidar and Orr, 1999). This evidence supports the model that phytochromes are in the inactive (Pr) state under intense far-red light circumstances, which prevents phytochromes from entering the nucleus. PIFs are thus unrestricted from regulation and activate the genes, including those involved in auxin and cytokinin biosynthesis or transport, needed for haustorium initiation (Furuhashi et al., 2021; Pan et al., 2022) (**Figure 2**).

Another component needed for forming the *Cuscuta* haustorium is physical contact with its host (Tada et al., 1996; Furuhashi et al., 2011). Several herbaceous and woody plant species have been described that can perceive mechanical cues from their environment and respond physiologically to change their growth or morphology by a process known as thigmomorphogenesis (Börnke and Rocksch, 2018). During the past decade, progress has been made in investigating the underlying molecular mechanisms of mechanoperception and thigmomorphogenesis in several plant species (Monshausen and Gilroy, 2009; Hamilton et al., 2015; Börnke and Rocksch, 2018; Mano and Hasebe, 2021), but how these thigmomorphogenesis signaling pathways are involved in *Cuscuta* haustorium organogenesis remains largely unknown. Here, we propose a hypothetical model based on our current understanding of non-parasitic plant models (**Figure 2**). Ion channels and receptor-like kinases are examples of mechanosensory proteins (Monshausen and Gilroy, 2009; Hamilton et al., 2015; Börnke and Rocksch, 2018) that may be activated by a physical contact signal with hosts. Plasma membrane-localized mechanosensitive calcium channels, like Mid1-complementing activity (MCA), trigger Ca^2+^ influx, cytosolic Ca^2+^-dependent signaling, and activate the expression of downstream genes possibly through Ca^2+^ sensors, such as calmodulin or calcium-dependent protein kinases (Nakagawa et al., 2007; Kurusu et al., 2013; Börnke and Rocksch, 2018; Mano and Hasebe, 2021). Another potential candidate is the mechanosensitive channel of small conductance (MscS) -like (MSL) family proteins, which are reported to be stretch-activated anion channels for Cl^−^ and cause alteration in the membrane potential (Haswell et al., 2008; Maksaev and Haswell, 2012; Mano and Hasebe, 2021). Receptor-like kinases (RLKs) might also play a role in triggering protein kinase cascades and altering Ca^2+^ signaling (Humphrey et al., 2007; Shih et al., 2014; Börnke and Rocksch, 2018), which in turn may affect downstream gene transcription, change hormone status and induce haustorium formation (**Figure 2**).

### 3.2 Adhesion phase (attached haustorium)

In the adhesion phase, prehaustoria continue to grow toward the host and form adhesive disks (also known as holdfasts or upper haustorium). The *Cuscuta* haustorial epidermal cells in contact with the host proliferate anticlinally, elongate, and differentiate into secretory holdfast cells. These holdfast cells can secrete adhesive glue containing de-esterified pectins to secure the haustorium on their host’s surface and seal the gap between themselves and their host (**Figure 1F**) (Vaughn, 2002).

### 3.3 Penetration phase (invading haustorium)

Once a haustorium securely attaches to its host, the haustorium development enters the penetration phase (also known as intrusive phase), and the file and digitate cells in endophyte primordium continue to divide and form inner haustorial cells. These cortex-originated inner haustorial cells then penetrate through the epidermis and cortex of their host tissues. At the tip of the penetrating haustorium, the inner haustorial cells elongate and differentiate into searching hyphae (Vaughn, 2003). Searching hyphae continue invading through the apoplastic region of host cortex tissues and searching for host vasculature *via* tip growth (**Figure 1G**).

### 3.4 Vascular connection phase (mature haustorium)

The final stage of developing a functional haustorium is establishing a successful vascular connection between host and parasite. The searching hyphae continue tip growth to seek host vascular tissues. Once the searching hyphae reach the host vascular cells, they form interspecies plasmodesmata connections at the tip of the searching hyphae. These searching hyphae then convert their cell identity depending on the type of cells they contact. For instance, the searching hyphae that make contact with host xylem cells will obtain xylem identity and become xylic hyphae (Vaughn, 2006). The xylic hyphae differentiate into xylem conductive elements and form xylem bridges connecting the xylem system between the host and the parasite (**Figure 1H**). On the other hand, the searching hyphae that make contact with host phloem sieve elements will obtain phloem identity and become phloic hyphae (also known as absorbing hyphae) (Vaughn, 2006). The phloic hyphae differentiate into phloem-conductive elements with finger-like protrusions connecting the phloem system between the host and the parasite. The mechanisms involved in the transition of cell identity are currently unknown. However, based on the discovery of mRNA, small RNA, and small peptides exchanged between host and parasite *via* haustorial connection (Kim et al., 2014; Kim and Westwood, 2015; Shahid et al., 2018), we propose a potential hypothesis that these exchanged signals likely function as signals to facilitate cell identity conversion, which will be of interest for future investigation.

## 4 *Cuscuta* as a model for haustorium development in stem holoparasitic plants

Compared with our recent advances in understanding root parasitic plant haustorium development, our knowledge of haustorium development and parasitism behaviours on stem parasitic plants is relatively limited. According to an increasing number of research reports, the required signals for inducing haustorium formation are different, the parasitism behaviours between root and stem haustorium formation are distinct, and the mechanisms behind these phenomena are still not completely understood. Therefore, good model organisms representing stem holoparasitic plants will help investigate how parasitic plants evolved to have different strategies to effectively attach to various above-ground organs of their host. The *Cuscuta* genus is one of the most well-studied stem parasitic plants and here we summarized why they are the popular choices for scientists in this field.

### 4.1 Advantage 1: Distinct mechanisms involved in haustorium induction

A stem parasitic plant-specific model system is required because the mechanisms involved in haustorium induction differ between root and stem parasitic plants. For example, many studies indicate that detecting quinones or phenolics from the hosts, known as haustorium-inducing factors (HIFs), is a critical factor in inducing haustorium development for root parasitic plants, like those seen in the Orobanchaceae (Chang and Lynn, 1986; Goyet et al., 2019). On the other hand, stem parasites use tactile stimuli and light signals for haustorium induction (Tada et al., 1996; Furuhashi et al., 2011). These different factors required for triggering haustorium formation show the distinct strategies needed to adapt to underground and aboveground parasitism. For underground parasitism, physical contact and light signals would not be effective searching mechanisms for host root systems surrounded by soil. Consequently, the ability to detect host-specific HIFs would be the primary criteria for root parasitic plant haustorium induction. Therefore, using *Cuscuta* species for studying stem parasitic plant haustorium organogenesis could help us understand the unique mechanisms deployed in aboveground parasitism.

### 4.2 Advantage 2: Special parasitizing behaviors: Autoparasitism, hyperparasitism, cross-organ parasitism

Besides serving as a sound system for studying the distinctive mechanisms involved in haustorium initiation, *Cuscuta* species also perform some unique parasitizing behaviours that are currently underinvestigated, including hyperparasitism (Wilson and Calvin, 2017), autoparasitism (Krasylenko et al., 2021), and cross-organ parasitism (Jhu and Sinha, 2022). These unique parasitizing behaviours among multiple parasitic plants have been previously reviewed (Krasylenko et al., 2021; Jhu and Sinha, 2022). Therefore, here, we focus on providing clear definitions with concise discussion and then propose hypotheses to understand why *Cuscuta* species evolved these unique parasitizing behaviours.

Hyperparasitism indicates the phenomenon of a parasitic plant parasitizing another parasitic plant. For stem parasites that attach to the aerial part of their host, this phenomenon is also known as epiparasitism because they grow “on top of” another parasite (Wilson and Calvin, 2017). Several previous studies have reported *Cuscuta* hyperparasitism on other parasitic plants. Hyperparasitism can happen between species or within the same species (Hawksworth, 1996). When a parasitic plant parasitizes the same parasitic plant species, this is known as autoparasitism. One specific type of autoparasitism is when the parasitic plants form functional haustoria on themselves, which is also known as self-parasitism (Fineran, 1965). In *Cuscuta* species, both autoparasitism and self-parasitism are commonly observed (Krasylenko et al., 2021). This is a distinctive parasitism strategy compared with several facultative or obligate root parasitic plants, which have self- and kin-recognition mechanisms to avoid forming haustoria on themselves or their similar relatives.

Deploying hyperparasitism and autoparasitism strategies might be the evolutionary consequence of adapting to aboveground parasitism. One hypothesis proposed by McLuckie is that self-parasitic haustoria in *Cassytha* or *Cuscuta* species might facilitate water conduction and long-distance transport (McLuckie, 1924). In addition, based on the observation that *Cuscuta* species also often form attachments to non-biological materials (Bernal-Galeano et al., 2022), we propose that the formation of these non-conductive haustoria or self-parasitic haustoria might both serve as physical support for *Cuscuta* to spread over a wider area and reach longer distances, which increases the possibility of finding new hosts and is therefore, beneficial for parasite survival.

Cross-organ parasitism refers to the phenomenon of a parasitic plant forming nonconventional haustorial attachments with other host organs (Jhu and Sinha, 2022). For example, *C. campestris* is known as a parasitic stem plant but has been reported to be able to form haustoria on tomato seedling roots (Jhu and Sinha, 2022). The reason why *Cuscuta* species evolved to have the ability to parasitize different organs of their host is still a mystery. However, the mechanisms of haustorium induction and host-specificity might contribute to these characteristics. Previous studies indicate that parasitic plants that can form self-parasitic haustoria, like *Cuscuta*, *Cassytha* and some hemiparasitic Orobanchaceae, can form haustoria without depending on detecting host-specific HIFs and are also more likely to show hyperparasitism (Krasylenko et al., 2021). These parasites usually also have a wider host range. To parasitize different host species, which might have different anatomical structures, having a higher degree of haustorium plasticity and HIF-independent haustorium induction might be criteria to adapt to different host structures and form successful haustorial connections with them. Therefore, this higher degree of haustorium plasticity might also confer on *Cuscuta* the ability to form haustoria on different organs of their hosts.

### 4.3 Advantage 3: Whole genome and abundant transcriptome data are available

Another advantage of using *Cuscuta* species as a model organism is the availability of whole genome information and different types of transcriptome data. The genomes of *C. campestris* and *C. australis* were both published in 2018 (Sun et al., 2018; Vogel et al., 2018). The *Cuscuta* transcriptome profiles in different developmental stages and tissue types are readily available (Ranjan et al., 2014; Jhu et al., 2021; Jhu et al., 2022; Bawin et al., 2022). *Cuscuta* mobile mRNAs and miRNAs that can transfer into the host plants are also reported (Kim et al., 2014; Shahid et al., 2018). These resources will facilitate research on *Cuscuta* development and the interaction between hosts and *Cuscuta* plants.

### 4.4 Advantage 4: Easy propagation and *in vitro* haustorium system

Another key criterion that makes an organism a good model organism is the ability to propagate or reproduce easily in vast numbers. *Cuscuta* species can quickly propagate through vines and produce many fruits and seeds, which can remain viable for more than ten years (Lanini and Kogan, 2005; Goldwasser et al., 2012; Masanga et al., 2022). These characteristics make them excellent model organisms. In addition, as mentioned in previous sections, many studies have shown that far-red light signals and physical contacts can trigger *Cuscuta* haustorium formation (Furuhashi et al., 1995; Tada et al., 1996; Furuhashi et al., 1997; Haidar and Orr, 1999). Therefore, several *in vitro* methods of growing *Cuscuta* or inducing haustorium formation without hosts have also been developed simultaneously (Kaga et al., 2020; Jhu et al., 2021; Bernal-Galeano et al., 2022). These methods allow us to investigate the function of genes in haustorium organogenesis more effectively and remove the influence or variations created by host conditions.

### 4.5 Major limitations: Lack of efficient transformation systems to obtain stable *Cuscuta* transgenic lines

To study the function of genes, generating mutants or transgenic plants with specific gene knockouts or gene overexpression is one critical approach. Unfortunately, the current major limitation of using *Cuscuta* species as a model is lack of efficient transformation methods to obtain stable transgenic lines. Several methods for producing successful transformation events have been published in different *Cuscuta* species, including *Cuscuta* *trifolii, Cuscuta reflexa*, and *Cuscuta europaea* (Borsics et al., 2002; Švubová and Blehová, 2013; Lachner et al., 2020).* *However, regenerating the transformed cells into calli and whole plants is still an unsolved issue. Developing a transformation system to generate stable transgenic *Cuscuta* plants will be a significant breakthrough technology.

At the same time, scientists have been developing different tools and methods for functional gene studies to bypass and overcome the current transformation obstacles. Based on previous studies, *Cuscuta* haustoria not only transport water and nutrients but also can transport small RNAs, messenger RNAs and small peptides (Kim et al., 2014; Kim and Westwood, 2015; Shahid et al., 2018; Wu, 2018; Johnson and Axtell, 2019). Therefore, host-induced gene silencing (HIGS) is a commonly used method to knock-down specific gene expressions in *Cuscuta* by attaching them to transgenic hosts that carry RNA interference (RNAi) constructs targeting *Cuscuta* genes (Albert et al., 2006; Alakonya et al., 2012; Jhu et al., 2021; Jhu et al., 2022). This method has been used widely in the *Cuscuta* research field to conduct functional analysis on genes of interest.

## 5 Conclusions

This review summarises the four major developmental stages of haustorium organogenesis in *Cuscuta* species. We organize the current understanding of haustorium induction by light signals and physical contacts and propose a potential pathway for haustorium formation. The detailed mechanisms of phytochrome signalling, hormone regulation, and thigmomorphogenesis in *Cuscuta* haustorium development are still elusive and will be of interest for future research. We also discuss the four significant advantages of using *Cuscuta* species as model organisms for haustorium development research, including the unique haustorium induction mechanisms, parasitizing behaviours, abundant genetic resources, and easy propagation. The current major limitation is absence of efficient transformation systems to obtain stable *Cuscuta* transgenic lines. However, host induces gene silencing has been widely used to overcome this limitation. With several reports on successful transformation events published recently from different research groups, we are optimistic that stable *Cuscuta *transformation systems might be established in the near future.

## Author contributions

M-YJ wrote the initial draft and prepared the figures. M-YJ and NRS both revised and approved the final manuscript. All authors contributed to the article and approved the submitted version.
